# Recent trends in airway management

**DOI:** 10.12688/f1000research.21914.1

**Published:** 2020-05-13

**Authors:** Basem B. Abdelmalak, D John Doyle

**Affiliations:** 1 Department of General Anesthesiology, Cleveland Clinic, 9500 Euclid Avenue, E-31, Cleveland, OH, 44195, USA; 2 Department of Outcomes Research, Cleveland Clinic, 9500 Euclid Avenue, E-31, Cleveland, OH, 44195, USA; 3 Department of General Anesthesiology, Cleveland Clinic Abu Dhabi, Abu Dhabi, United Arab Emirates

**Keywords:** Airway Management, Lower Airway Management, the physiologically difficult Airway, Videolaryngoscope, Jet ventilation

## Abstract

Clinical airway management continues to advance at a fast pace. To help update busy anesthesiologists, this abbreviated review summarizes notable airway management advances over the past few years. We briefly discuss advances in video laryngoscopy, in flexible intubation scopes, in jet ventilation, and in extracorporeal membrane oxygenation (ECMO). We also discuss noninvasive ventilation in the forms of high-flow nasal cannula apneic oxygenation and ventilation and nasal continuous positive airway pressure (CPAP) masks. Emerging concepts related to airway management, including the physiologically difficult airway and lower airway management, new clinical subspecialties and related professional organizations such as Anesthesia for Bronchoscopy, the Society for Head and Neck Anesthesia, and fellowship training programs related to advanced airway management are also reviewed. Finally, we discuss the use of checklists and guidelines to enhance patient safety and the value of large databases in airway management research.

## Introduction

Airway management is an essential part of what anesthesiologists do every day and, consequently, is a skill in which every anesthesiologist strives for expertise. No wonder it has been the subject of much research and innovation. This synopsis is aimed at summarizing some of the recent trends and advances in airway management, research, and innovation.

## Video laryngoscopy

**Figure 1.**
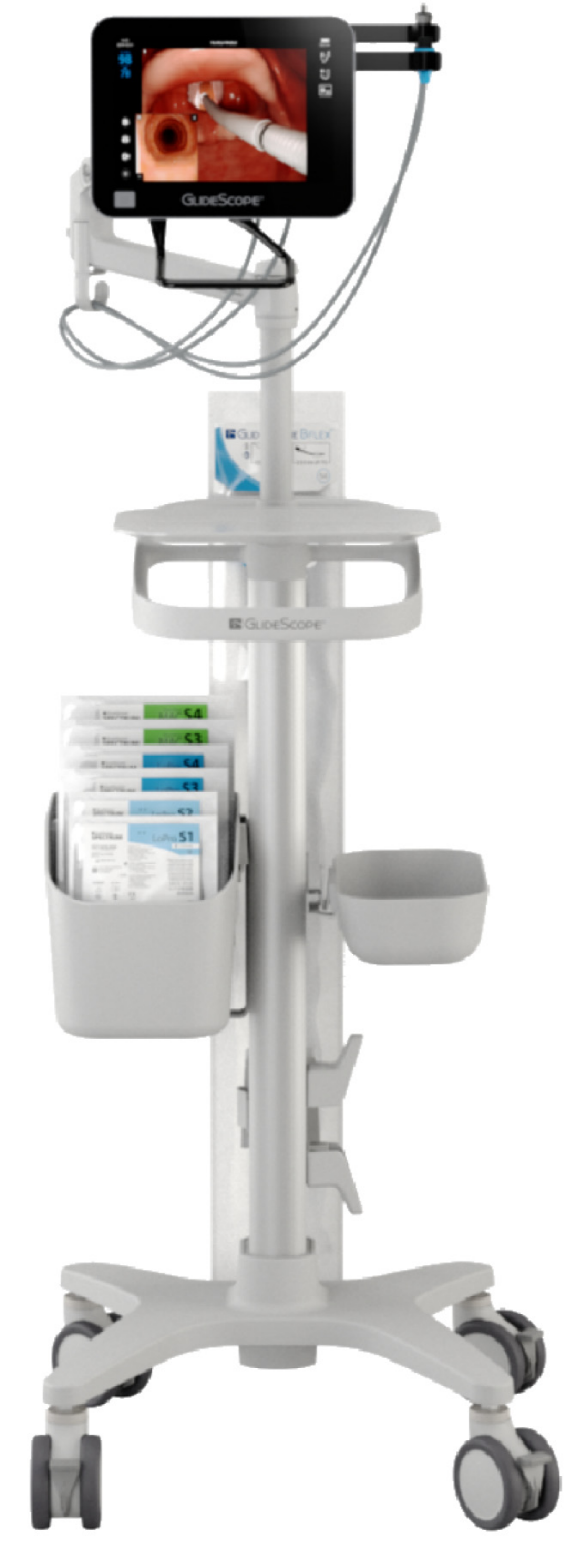
The GlideScope Core™ airway visualization workstation. The GlideScope Core™ airway visualization workstation offers a 10 inch high-resolution color touch-screen display with picture-in-picture capability to allow simultaneous laryngoscopy and bronchoscopy. Still images and videos can be captured for later review. An adjustable arm enables one to position the monitor for optimal viewing. Other features include the display of patient SpO _2_ and 180 degree image rotation. This image has been reproduced with permission from Verathon Inc.

In video laryngoscopy, a form of indirect laryngoscopy, the operator does not view the larynx directly but instead views the larynx indirectly using a tiny imaging device, such as a microminiature CCD camera. Video laryngoscopes vary with blade design and angulation, may be channeled or non-channeled, and are often portable. Popular videolaryngoscopes include the GlideScope family of products (Verathon Inc., Bothell WA, USA), C-MAC (Karl Storz SE & Co. KG, Tuttingen, Germany), Pentax (Pentax Medical, Akishima-shi, Tokyo, Japan), and McGrath Products (Medtronic Inc., Minneapolis, MN, USA), and many others. One particularly interesting new product is the GlideScope Core™, which offers picture-in-picture imaging to combine video laryngoscope and video bronchoscope capability into a single unit ( Figure 1). For reviews of video laryngoscopy, the interested reader is directed to recent open access publications by Parotto and Cooper ^1^ and Doyle ^2^. While these advanced devices have certainly improved laryngeal visualization and intubation success, safe airway management still remains a challenge, with failures ^3^ and complications still occasionally occurring ^4^.

## Flexible intubation scopes

Flexible intubation scopes have been available for decades and are considered by many to be the most versatile tool in navigating the anatomically difficult airway. Uses include steering past airway masses, passing through airways narrowed from edema, tumors, or hematomas, nasally intubating patients with severe trismus, or entering a tracheotomy to ensure that no false passage is present. Flexible intubation scopes are especially well tolerated in "awake" intubation procedures, which might be more aptly described as intubation under topical anesthesia while the patient is sedated and breathing spontaneously.

In recent years, flexible intubation scopes have evolved from using fiberoptic technology to using video chip technology (albeit still referred to as fiberoptic owing to familiarity with the accepted term) with greatly improved image quality. Additionally, with increased concern regarding cleaning and decontamination ^5^, as well as the costs of processing and ongoing maintenance, disposable flexible intubation scopes have become available. Examples include products from Karl Storz, Ambu (Copenhagen, Denmark), and Verathon Inc.

The importance of awake intubation is highlighted by the recently published Difficult Airway Society (DAS) guideline ^6^ and similar initiatives. While such guidelines are obviously valuable, these practice recommendations are essentially a consensus statement by airway experts directed at the average anesthesiologist and may be potentially problematic in that not all of the recommendations have been rigorously vetted or backed by extensive research. Additionally, some recommendations may be impractical in limited resource settings. Possible examples include the use of remifentanil sedation, use of 10% lidocaine, use of high-flow nasal oxygen (HFNO) delivery, or calling for the presence of a second anesthetist to assist with elective awake intubation. Awake intubation failures have been reported in certain instances, such as inadequate topicalization, airway obstruction during topicalization, and oversedation leading to airway obstruction ^4^. Proper technique, training, and practice may mitigate many of these challenges.

## The Ventrain system

Although mechanical ventilation is typically achieved using 6 to 8 mm internal diameter (ID) cuffed endotracheal tubes (ETTs), there are circumstances where this arrangement is unsatisfactory. For example, in some laryngeal surgeries, in order to provide the surgeon with an unobstructed glottic view, a narrow-diameter catheter (e.g. Hunsaker catheter) is used ( Figure 2a). This high-resistance catheter is driven by a high-pressure gas source. The technique is commonly referred to as subglottic jet ventilation in this setting ( Figure 2b), in contrast to supraglottic jetting ( Figure 3), when the jetting needle is attached to the eye-piece end of the rigid laryngoscope. Another example is the “cannot intubate, cannot ventilate” (CICV) airway situation, where a very narrow-bore high-resistance catheter is placed into the trachea percutaneously (percutaneous transtracheal jet ventilation or TTJV). Note that in such situations expiration during ventilation occurs passively through elastic recoil of the lungs and requires some degree of airway patency.

**Figure 2a.**
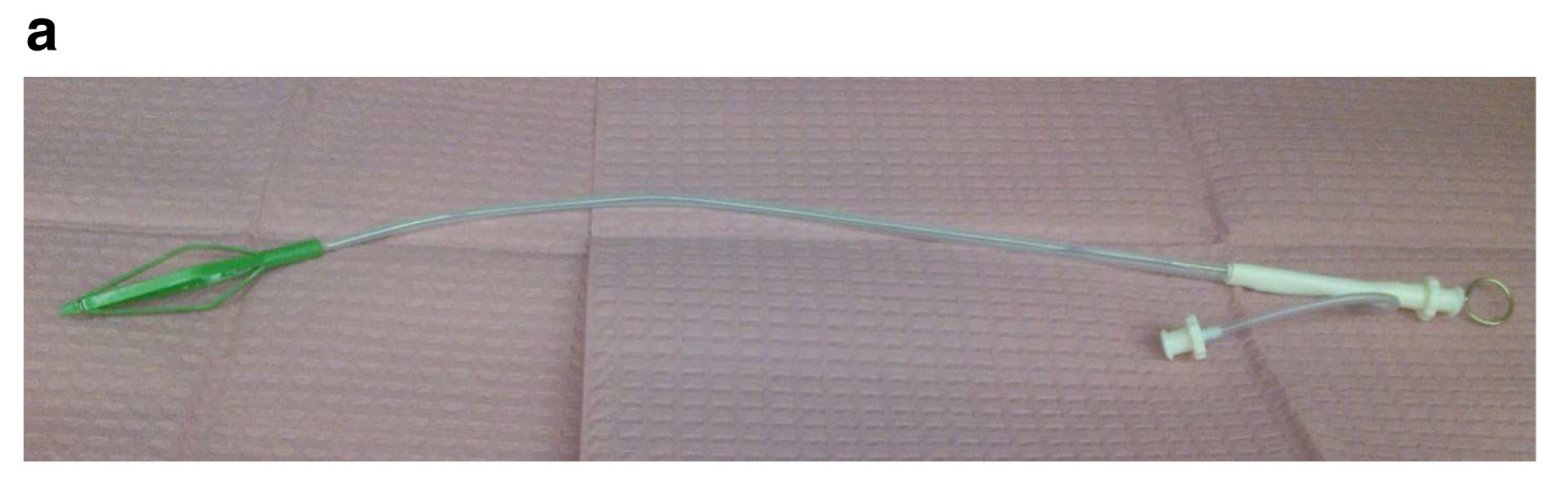
Hunsaker catheter. This figure has been reproduced with permission from Cleveland Clinic Center for Medical Art & Photography © 2020. All rights reserved.

**Figure 2b.**
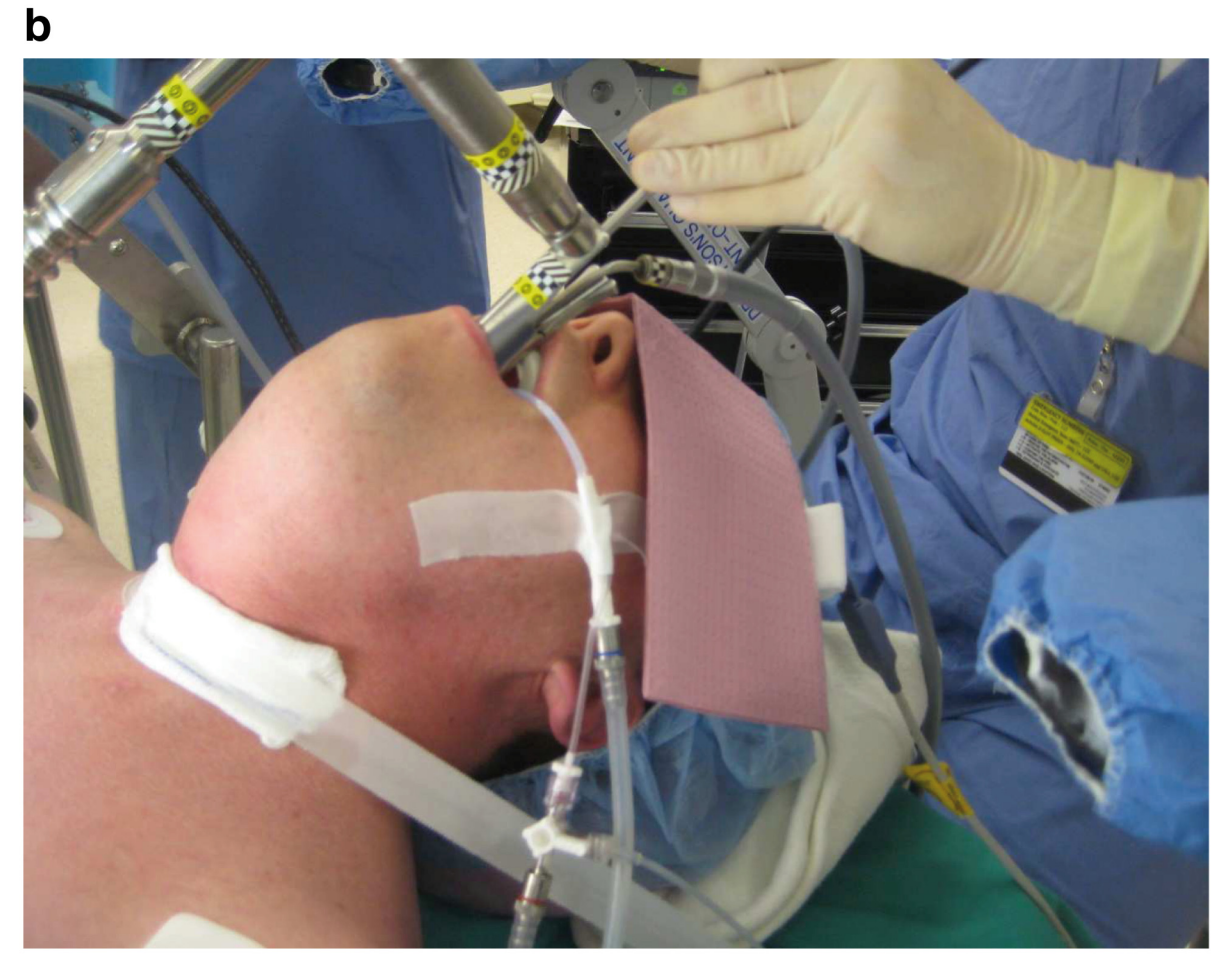
Subglottic jetting using Hunsaker catheter (see Figure 2a). Please note that the catheter sideport is attached to the airway pressure monitoring tube and, through the stopcock, it can be connected temporarily to an EtCO _2_ monitor showing a capnogram. This figure has been reproduced with permission from Cleveland Clinic Center for Medical Art & Photography © 2020. All rights reserved. Written informed consent was obtained from the patient for the use and publication of this clinical image.

**Figure 3.**
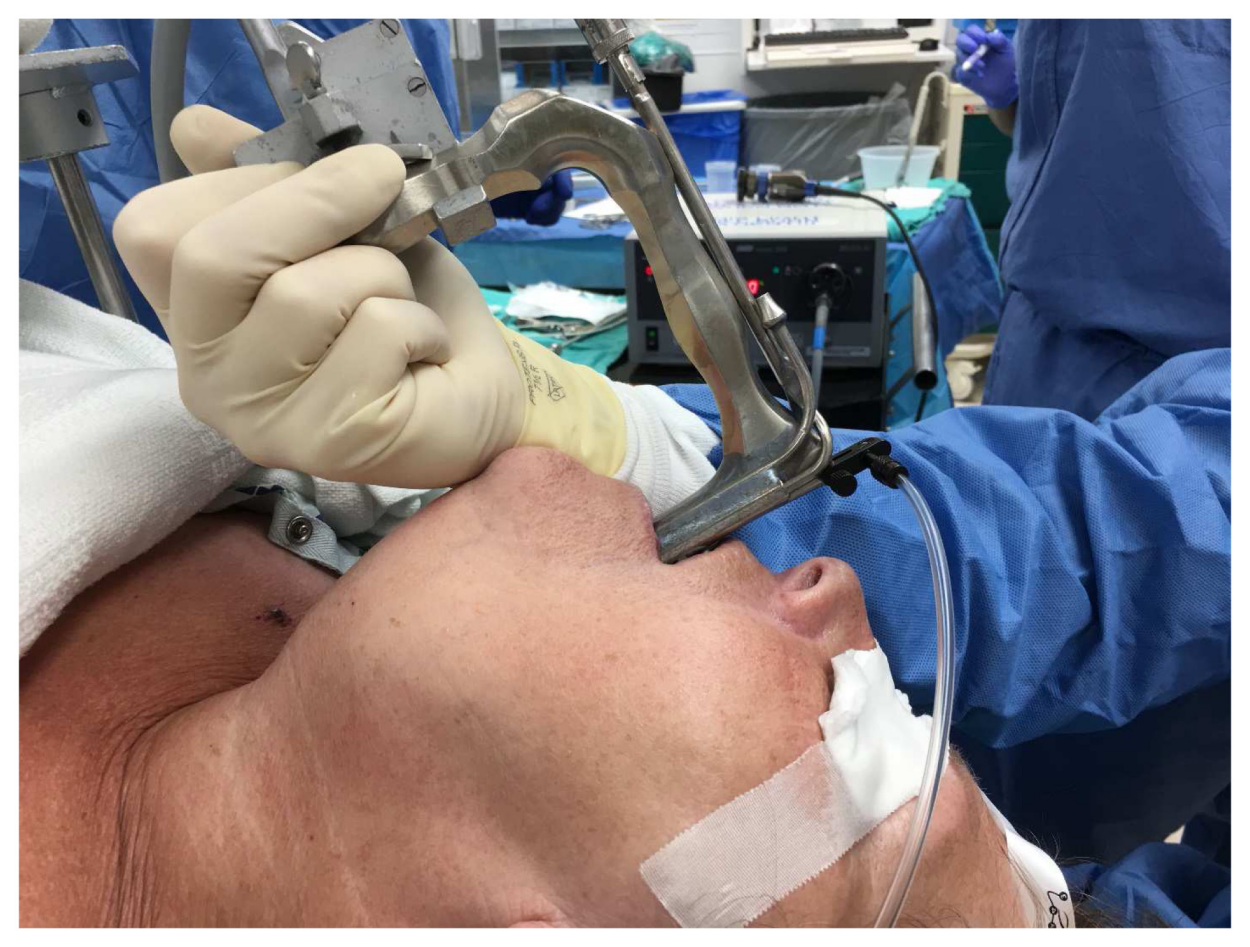
Supraglottic jetting. The jetting needle is attached to the eye piece of the suspension laryngoscopy scope. This figure has been reproduced with permission from Cleveland Clinic Center for Medical Art & Photography © 2020. All rights reserved. Written informed consent was obtained from the patient for the use and publication of this clinical image.

However, a recently introduced apparatus now exists that supports active expiration, even in fully obstructed airways, via a narrow-bore tracheal catheter. The device, known as the Ventrain system (Ventinova Inc., Eindhoven, The Netherlands), provides complete ventilation using a mere 2 to 3 mm catheter. This system uses active expiration, drawing on the well-known Bernoulli Principle, and avoids extreme intrapulmonary pressures and the associated pulmonary (baro)trauma that occasionally complicates jet ventilation. This product has been a true lifesaver by providing full ventilation in CICV situations in both babies and adults ^7– 10^.

**Figure 4.**
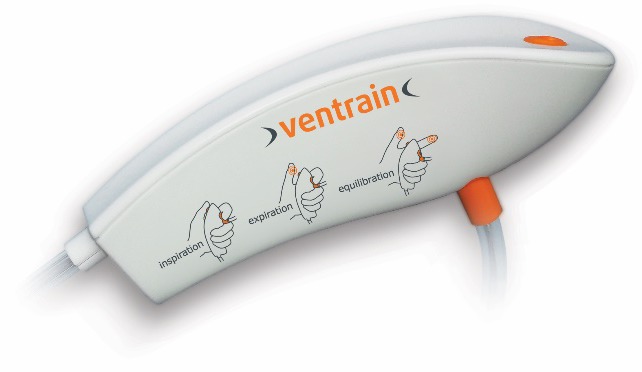
The Ventrain® device. This simple-to-operate manual ventilator is operated using one’s thumb and index finger. It features active expiration based on the Bernoulli Principle, allowing ventilation through small-bore tubes. In addition to inspiratory (positive pressure) and expiratory (negative pressure) modes of operation, an equilibration (safety) mode is available where no significant positive or negative pressure is present at the tip of the attached ventilation catheter. The figure and text have been reproduced with permission from Ventinova Medical, Eindhoven, The Netherlands.

**Figure 5.**
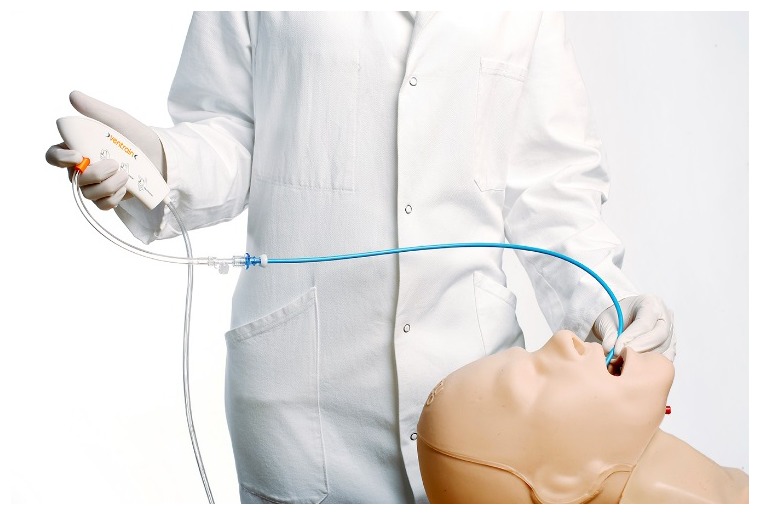
Illustration of the Ventrain® used in conjunction with a small-bore airway catheter, such as those used to exchange endotracheal tubes or those used to assist with tracheal extubation. In addition to providing a means to facilitate reintubation, such airway catheters can also be used with the Ventrain® to maintain ventilation and oxygenation until a definitive airway is established. The figure and text have been reproduced with permission from Ventinova Medical, Eindhoven, The Netherlands.

**Figure 6.**
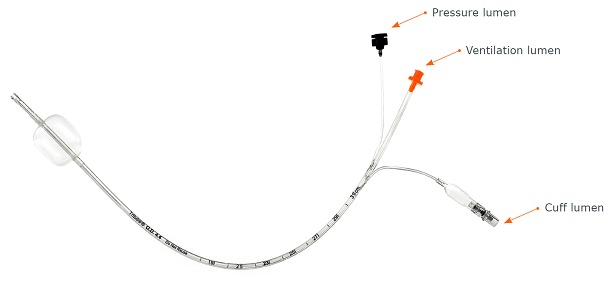
The Tritube®. The Tritube® is a narrow-bore cuffed endotracheal tube with inner and outer diameters of 2.4 mm and 4.4 mm, respectively. A Murphy eye is featured at the distal end. The ventilation lumen is attached to the Ventrain®, while an inflatable cuff seals the airway. A pressure measurement lumen permits continuous intratracheal pressure measurements. This device may be useful in both elective and emergency airway settings. The figure and text have been reproduced with permission from Ventinova Medical, Eindhoven, The Netherlands.

As Figure 4 and Figure 5 illustrate, the Ventrain ventilator is a hand-held, manually operated device designed specifically for patient ventilation via narrow-bore catheters such as the Tritube® cuffed ETT ( Figure 6). This design has been shown to provide a minute ventilation exceeding 7 L/minute with oxygen flows of 15 L/minute while using ventilation catheters with internal diameters as small as 2 mm ^11^.

In CICV situations during upper airway surgery, use of the Ventrain system has both improved surgical exposure and avoided the potential need for tracheostomy surgery. Borg *et al*. ^12^ were the first to describe the use of the Ventrain system during laryngoscopy in a patient with an exophytic glottic tumor presenting with stridor. The authors placed a 2 mm ID transtracheal catheter through the cricothyroid membrane under local anesthesia and then connected the catheter to the Ventrain at an oxygen flow of 15 L/minute. General anesthesia was then started, with the patient ventilated using the Ventrain. The procedure allowed biopsy samples to be obtained that were needed for the patient’s pathology to be determined.

Onwochei *et al*. ^13^ described a method to avoid a tracheostomy in a 49-year-old woman suffering from “post radiotherapy laryngeal fixation and transglottic stenosis”. To provide needed airway dilation, the authors began with awake flexible scope intubation, followed by the transtracheal insertion of a Cricath flexible 2 mm needle cricothyrotomy catheter. Ventilation was achieved using the Ventrain system. Fearnley *et al*. ^14^ describe a similar case involving upper airway obstruction due to post-radiation fibrosis.

Finally, in seven ENT patients, Kristensen *et al*. ^15^ used the Ventrain system along with a special 2.4 mm ID cuffed ETT (Tritube®) that allows intratracheal pressure monitoring. In all patients, adequate ventilation was achieved, with intratracheal pressures ranging between 5 and 20 cm H _2_O. The authors noted that this combination provided an “unprecedented view of the intubated airway during oral, pharyngeal, laryngeal or tracheal procedures” and further noted that the method has the “potential to replace temporary tracheostomy, jet-ventilation or extra-corporal membrane oxygenation in selected patients”.

Where a hand-operated ventilation technique is onerous, clinicians may wish to use the newly launched automatic ventilator Evone instead. Details are available at https://www.ventinovamedical.com/evone/.

## Extracorporeal membrane oxygenation in the management of the critical airway

In drastic cases, extracorporeal membrane oxygenation (ECMO) may sometimes be used in critical airway management. Holliday and Jackson ^16^ reported on the use of ECMO in a severely intoxicated patient with a life-threatening airway obstruction that occurred despite the presence of an ETT. A large tracheal food bolus situated beyond the ETT tip was revealed using bronchoscopy. After the institution of ECMO, the obstructing material was removed via rigid bronchoscopy. Reviews by Hoetzenecker *et al*. ^17^ and Meng *et al*. ^18^ provide a summary of the published ECMO experience in relation to airway surgery.

## Non-invasive ventilation and anesthesia

### High-flow nasal oxygenation

**Figure 7.**
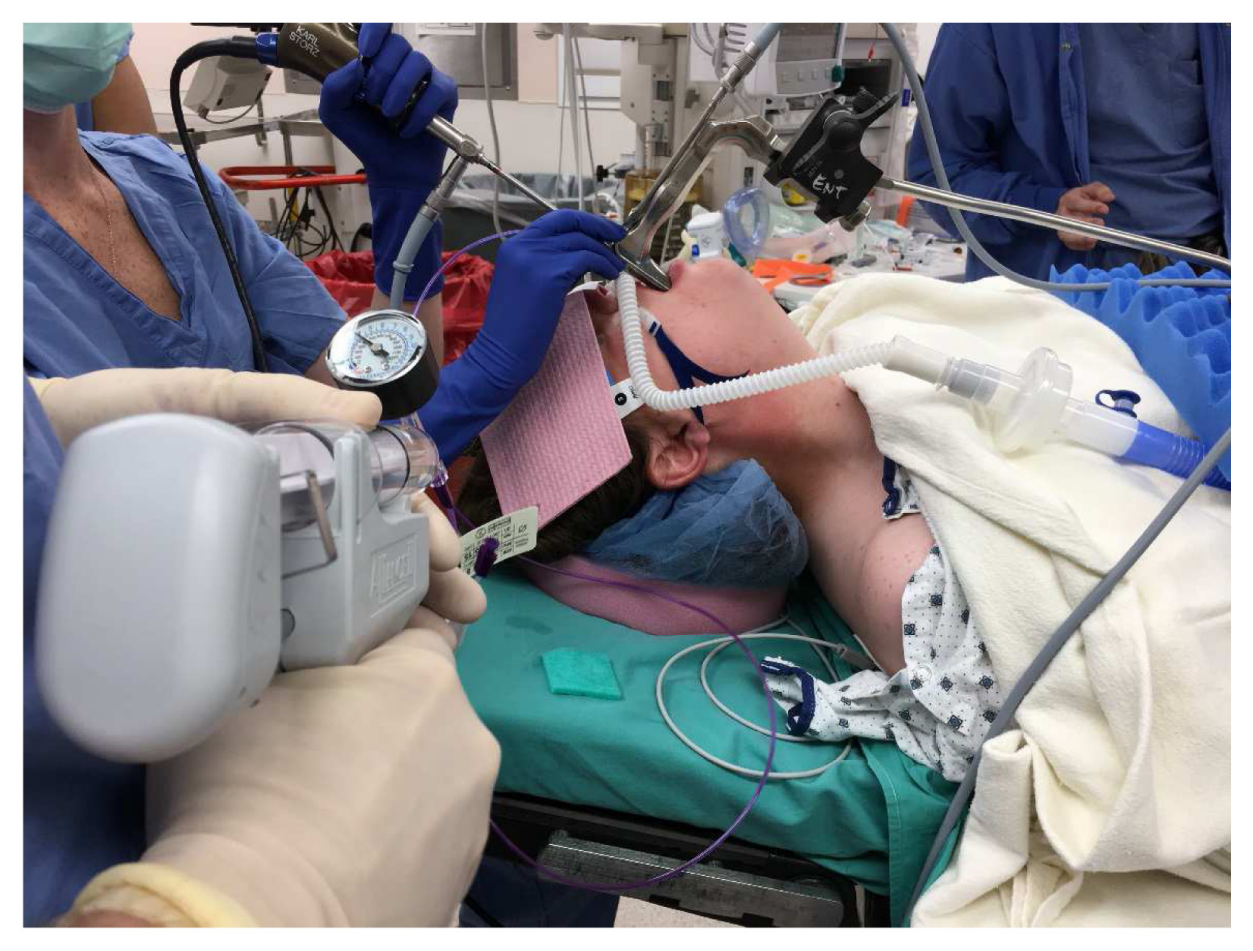
High-flow nasal oxygenation and ventilation technique is used during balloon dilation of subglottic stenosis in an anesthetized and paralyzed patient. This figure has been reproduced with permission from Cleveland Clinic Center for Medical Art & Photography © 2020. All rights reserved. Written informed consent was obtained from the patient for the use and publication of this clinical image.

HFNO, sometimes known as transnasal humidified rapid-insufflation ventilatory exchange (THRIVE), is a method of apneic oxygenation and ventilation that has recently been introduced to the operating room for use during the management of the difficult airway as well as for shared airway surgery, rapid sequence inductions, and management of morbidly obese patients ^19, 20^. This technique has become the “go to” technique for many clinicians, especially in laryngeal surgery ( Figure 7) ^21, 22^. However, with pre-oxygenation and during intubation, comparative studies against the use of an ordinary facemask had notable limitations in their design. In some instances, HNFO is used continuously both before and during intubation and compared against the use of a facemask during the preoxygenation phase only, as the facemask is removed during intubation. This difference dictates that further studies need to be undertaken to provide additional clarity.

### Nasal continuous positive airway pressure mask

A recently introduced nasal continuous positive airway pressure device (SuperNO2VA™, Vyaire Medical, Yorba Linda, CA) was found to be superior to nasal cannula when used for GI procedures performed under deep sedation ^23^. More research is needed to characterize its usefulness in perioperative airway management.

## The physiologically difficult airway

Challenges in airway management stem not only from dealing with anatomically abnormal airways but also from the pathophysiologic condition of the patient. Examples of such conditions include hypoxemia, hypotension, severe metabolic acidosis, and right ventricular failure. For instance, when hypoxemia is present because of causes such as atelectasis, pneumonia, ARDS, or pulmonary edema, special attention to preoxygenation, apneic oxygenation, and alveolar recruitment maneuvers apply. In some instances, patients may not be expected to tolerate even a very brief period of apnea and thus spontaneous ventilation should be maintained during the intubation process, such as the case in “awake intubation”. In the instance of hypotension, special attention to preserving venous return to the heart is imperative, since high ventilation pressures with resulting increases in intrathoracic pressure may worsen the situation by decreasing venous return. While fluid resuscitation in this setting will ordinarily increase circulating volume, venous return, and cardiac output, if the right heart cannot accommodate the increased volume (for example, as a result of right ventricular failure), this intervention may backfire. For a detailed discussion of pathophysiologic conditions impacting on airway management, the interested reader is directed to an open access review by Mosier *et al*. ^24^. In some of these situations, such as in patients with what is described by Abdelmalak *et al*. ^25^ as a “critical airway” (a difficult airway with impending failure), where the patient presents with stridor from a mass effect complicated by hypoxemia, awake intubation using sedation that preserves respiratory drive can be of immense value ^25^.

## Lower airway management

**Figure 8.**
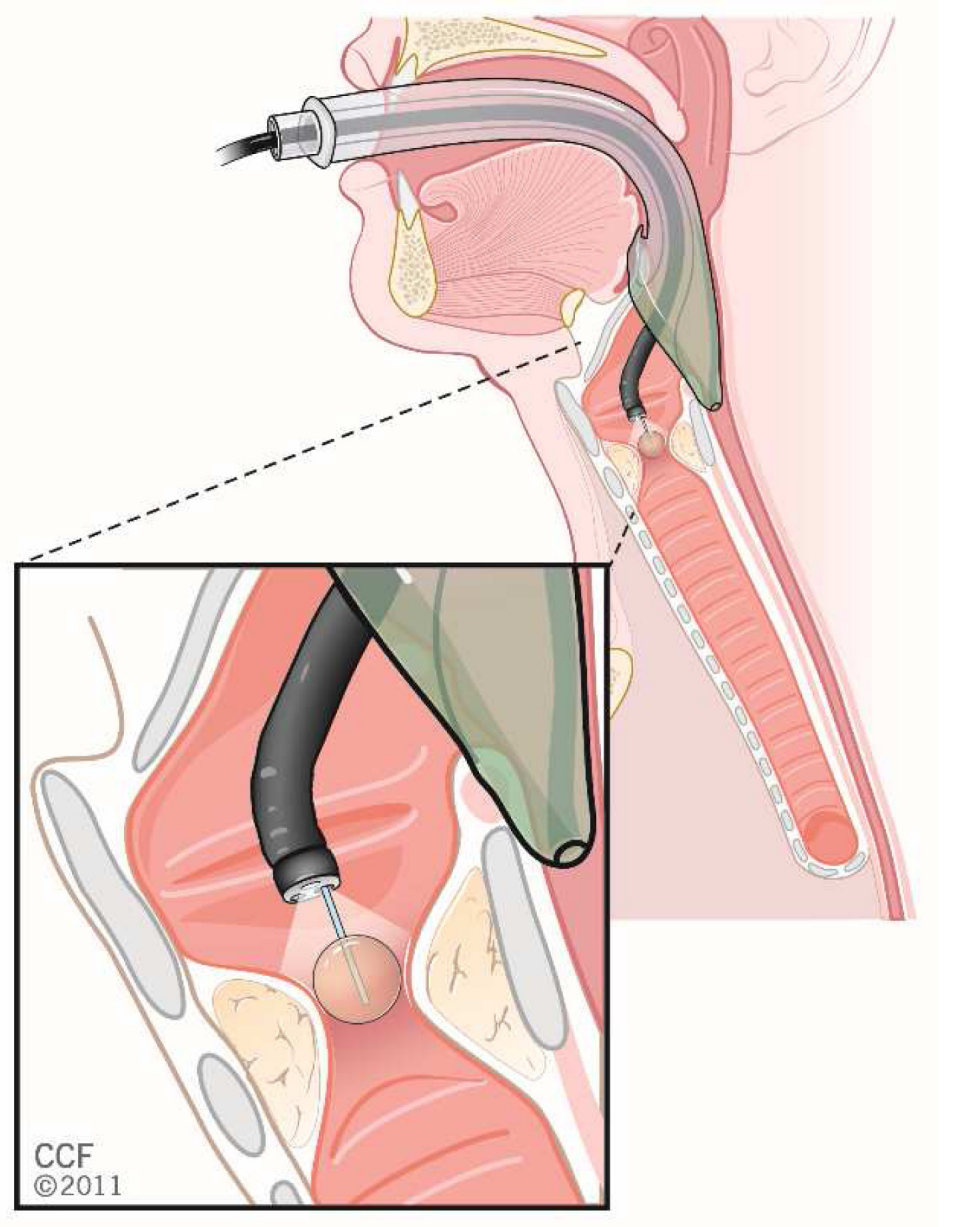
Illustration of how a supraglottic airway can be used to provide ventilation while also serving as a conduit for a flexible bronchoscope and balloon dilator used to dilate a subglottic stenosis. This image has been reproduced with permission from Cleveland Clinic Center for Medical Art & Photography © 2020. All rights reserved.

**Figure 9.**
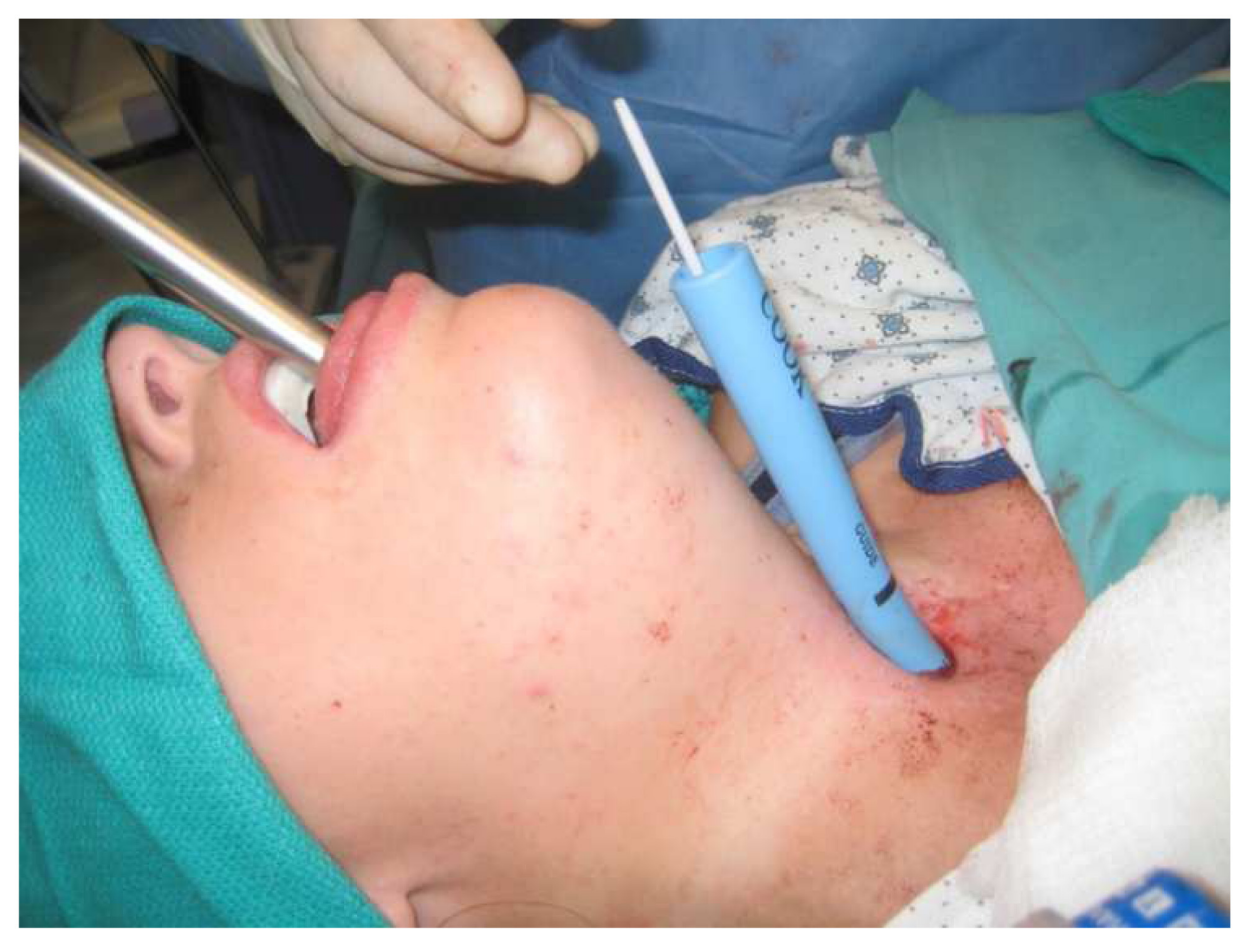
Illustration of how a rigid bronchoscope is used to access the upper airway while an old tracheostomy site is used to access a lower portion so as to re-establish the connection between the upper and lower portions after the connection had closed off because of granulation and fibrous tissue resulting from long-term tracheostomy use. Ventilation was accomplished with positive pressure ventilation with a semi-closed circuit through the rigid bronchoscope side port or through intermittent intubation of the old tracheostomy site, depending on the stage of the procedure. Alternatively, jet ventilation or high-flow oxygen apneic oxygenation and ventilation could have been used. This figure has been reproduced with permission from Cleveland Clinic Center for Medical Art & Photography © 2020. All rights reserved. Written informed consent was obtained from the patient for the use and publication of this clinical image.

Abdelmalak *et al*. ^26– 28^ have emphasized that managing "lower airway" problems such as tracheal stenosis, tracheoesophageal fistula, anterior mediastinal mass, tracheal rupture, bronchopleural fistula, etc. ( Table 1) can be just as challenging as managing traditional "upper airway" problems. They stress that lower airway problems come with their own interventions and tactics, including different modes of ventilation, different devices, and different strategies for providing anesthesia. This can be demonstrated by the remarkable growth in the field of interventional pulmonology, where pulmonologists have introduced new tools and technologies not only to diagnose and stage lung cancer using techniques such as endobronchial ultrasound (EBUS) and endobronchial navigational bronchoscopy (ENB) but also to treat it with ablation techniques. These interventional pulmonologists also manage many of these challenges through a flexible operating scope and/or rigid bronchoscope. Figure 8 illustrates the use of a supraglottic airway to enable subglottic stenosis dilation through a flexible bronchoscope, while Figure 9 shows the use of rigid bronchoscope in the management of a complex airway procedure during which ventilation was maintained by the use of a standard semi-closed circuit. Alternative techniques in this case might have been jet ventilation, intermittent apnea with or without intermittent intubation, and high-flow oxygen apneic oxygenation and ventilation. Such a variety of available techniques showcases the flexibility and adaptability of both interventional pulmonology and anesthesiology teams.

**Table 1.** Examples of lower airway challenges.

Category	Example
Subglottic stenosis	Idiopathic subglottic stenosis and granulomatosis with polyangiitis
Tracheostomy and Montgomery T-tube management	Urgent or emergent versus elective tracheostomy. Long-term management interventions for tracheostomy or T-tubes such as replacement, upsizing or downsizing, and decannulation
Endoluminal, extraluminal, or mixed central airway obstruction	Bronchogenic carcinoma, goiter, mediastinal mass, etc.
Tracheal disruption	Traumatic tear or tumor
Bronchial disruption	Dehiscence of lung transplant anastomosis
Tracheal resection surgeries	Certain types and degrees of tracheal stenosis or tumor invasion
Tracheoesophageal fistula	Esophageal or bronchogenic cancer complications versus traumatic fistula
Bronchopleural fistula	Post-thoracic surgery
Less-common conditions	Tracheomalacia, tracheal reconstruction after an airway fire

Reprinted with permission, Cleveland Clinic Center for Medical Art & Photography © 2018. All rights reserved.

## Societies, guidelines, and checklists

Increasingly, publications from different national and subspecialty societies for airway management in various patient populations (e.g. trauma patients, obstetrical patients, etc.) and clinical situations (e.g. extubation) have become available. Special mention should be made of recommendations from the ASA ( http://www.asahq.org), the DAS ( http://das.uk.com/) as well as helpful resources provided by the European Airway Management Society ( http://www.eamshq.net/), the Society for Airway Management ( http://samhq.com/), the National Tracheostomy Safety Project ( http://www.tracheostomy.org.uk/), and the Society for Head and Neck Anesthesia ( https://www.shanahq.com/). A cognitive aid viewed by many as helpful for difficult airway management, known as the Vortex Approach ( http://vortexapproach.org/), is based on the notion that there are only three non-surgical techniques for oxygen delivery (face mask, supraglottic airway, and ETT) and if a “best effort” at each of these three techniques is unsuccessful then emergency front-of-neck airway access (FONA) must be undertaken.

## Head and neck anesthesia and advanced airway management fellowships

Owing to the increased complexity of the airways encountered in the field of head and neck surgery requiring especially skilled anesthesiologists to safely manage the airway, some institutions are now offering head and neck anesthesia and advanced airway management fellowships. Such fellowships are offered at Stanford University, University of Michigan, Mount Sinai Medical Center, University of Toronto, and Montefiore Medical Center.

## Large database and observational trials

The availability of large clinical outcome databases from initiatives such as the Multicenter Perioperative Outcomes Group ( https://mpog.org) and the UK NAP4 National Audit Project ( https://www.nationalauditprojects.org.uk/NAP4_home) has provided important insights into airway management complications and has helped identify how improvements in airway management might be undertaken. As an example, the NAP4 study found that using a scalpel and bougie method for FONA had a much higher success rate as compared to the often-advocated cannula cricothyroidotomy approach ^27^. Nonetheless, this matter is still the subject of much debate. Insights such as this, as well as future randomized prospective studies intended (amongst other things) to verify consensus statements originally based on only limited empirical evidence, will help ensure that airway management will continue to be safe and improve with time. As a practical example, definitively establishing which is the best technique for managing patients with high aspiration risk, especially in the context of the rapid sequence intubation technique and cricoid pressure, is needed ^29^. Another example concerns identifying and modifying ergonomic (human factors) issues that contribute to airway management complications ^30^.
